# Patterns of microchromosome organization remain highly conserved throughout avian evolution

**DOI:** 10.1007/s00412-018-0685-6

**Published:** 2018-11-17

**Authors:** Rebecca E. O’Connor, Lucas Kiazim, Ben Skinner, Gothami Fonseka, Sunitha Joseph, Rebecca Jennings, Denis M. Larkin, Darren K. Griffin

**Affiliations:** 1 School of Biosciences, University of Kent, Canterbury, CT2 7NJ UK; 2 Department of Pathology, Cambridge University, Cambridge, CB2 1QP UK; 3 Cytocell Ltd, 3-4 Technopark Newmarket Road Cambridge, Cambridge, CB5 8PB UK; 4 Department of Comparative Biomedical Sciences, Royal Veterinary College, University of London, London, NW1 0TU UK

**Keywords:** Microchromosome, Evolution, Avian, Genome, Conservation

## Abstract

The structure and organization of a species genome at a karyotypic level, and in interphase nuclei, have broad functional significance. Although regular sized chromosomes are studied extensively in this regard, microchromosomes, which are present in many terrestrial vertebrates, remain poorly explored. Birds have more cytologically indistinguishable microchromosomes (~ 30 pairs) than other vertebrates; however, the degree to which genome organization patterns at a karyotypic and interphase level differ between species is unknown. In species where microchromosomes have fused to other chromosomes, they retain genomic features such as gene density and GC content; however, the extent to which they retain a central nuclear position has not been investigated. In studying 22 avian species from 10 orders, we established that, other than in species where microchromosomal fusion is obvious (*Falconiformes* and *Psittaciformes*), there was no evidence of microchromosomal rearrangement, suggesting an evolutionarily stable avian genome (karyotypic) organization. Moreover, in species where microchromosomal fusion has occurred, they retain a central nuclear location, suggesting that the nuclear position of microchromosomes is a function of their genomic features rather than their physical size.

**Electronic supplementary material:**

The online version of this article (10.1007/s00412-018-0685-6) contains supplementary material, which is available to authorized users.

## Introduction

The gross structure and organization of the genome of any individual species (both at a karyotypic level and in interphase nuclei) have broad functional significance. Specifically, the number and shape of chromosomes as well as the order of genes thereon can have an impact on the evolution, variation and phenotype of that species. In this regard, microchromosomes, present in many terrestrial vertebrates (but not mammals nor crocodilians), remain largely uncharacterized. Birds have a highly distinctive, ‘signature’ avian genome structure (karyotype) which is typically divided into ~ 10 macrochromosomes and ~ 30 evenly sized, morphologically indistinguishable microchromosomes (Christidis 1990; Masabanda et al. 2004; Griffin et al. 2007). The number and morphological similarity of avian microchromosomes are near-unique in nature, and they are impossible to distinguish using classical cytogenetic approaches. In fact, to date, although around 1000 karyotypes have been published for birds (a phylogenetic class that with ~ 10,500 extant species), these are all partial, with only 5–10 pairs of chromosomes readily identifiable. Nonetheless, this apparent degree of stability across a group with enormous phenotypic diversity is, at best, only inferred through a comparison of chromosome number from one bird to another. Exceptions to this rule include the *Falconiformes* (falcons) and the *Psittaciformes* (parrots), both of which have reduced diploid numbers relative to the norm, along with fewer microchromosomes. While this suggests evidence of chromosomal fusion (Nishida-Umehara et al. 2007; Nanda et al. 2007), the nature of these fusions is only recently being uncovered. Comparative genomics of avian macrochromosomes (up to chromosome 9) has been refined beyond karyotyping through the development of chromosome paints derived from amplification and fluorescence labelling of chicken macrochromosomes isolated by flow-cytometry (Griffin et al. 1999). Cross-species analysis on over 70 avian species from 15 different orders has revealed a remarkable lack of inter-macrochromosomal rearrangements. Some success has been achieved using microchromosomal paints (Lithgow et al. 2014), but this is limited to analysis of ‘pooled’ chromosomes (where each pool contains between 2 and 10 pairs of microchromosomes) due to the technical difficulties of separating individual microchromosomes by flow cytometry. A degree of success using a cross-species BAC (bacterial artificial chromosome) mapping approach has been reported, although until recently, this has been limited to closely related species, with 70% success rates reported using chicken BACs on turkey (*Mealeagris gallopavo*) (Griffin et al. 2008) reducing to 40–50% when tested on duck (*Anas platyrynchos*) (Fillon et al. 2007; Skinner et al. 2009). Investigation of the microchromosomes of the chicken and the zebra finch using a BAC mapping approach has also been reported by Völker et al. (2010), but this was achieved using the same specie hybridization with the comparative genomics performed in silico. As such, this approach can only be performed when both species have characterized BAC libraries, and even then, the very smallest microchromosomes (the so called ‘D-group’) have not been sequenced and would therefore not be amenable to this (to a BAC mapping) approach. We recently reported an approach to isolate BACs that hybridize in a universal manner across all bird species (Damas et al. 2017); however, to date, this has only been applied to five species (Damas et al. 2017; O’Connor et al. 2018b). To date, therefore, a meaningful comparative analysis of the majority of avian microchromosomes, in a range of species, has yet to be performed.

At a functional level, despite microchromosomes accounting for only 23% of the average avian genome size, they contain around 50% of genes and are thus highly GC-rich (average 48%) and gene-dense (McQueen et al. 1998; Smith et al. 2000; Habermann et al. 2001; Burt 2002; Warren et al. 2017). In the chicken (the most characterized of avian species), macrochromosomes range in size from ~ 23 to 200 Mb, but microchromosomes are, on average, only 12 Mb in length, the smallest being ~ 3 Mb (Hillier et al. 2004). Microchromosomes have been demonstrated to have a significantly higher recombination rate than macrochromosomes (Hillier et al. 2004; Backström et al. 2010) and, in the interphase nucleus, they appear to cluster in a central position, with the macrochromosomes occupying the nuclear periphery (Habermann et al. 2001; Federico et al. 2005; Skinner et al. 2009). Whether this is a function of their small physical size, or the fact that microchromosomes have a greater gene density (thereby more able to access the transcriptional machinery), is still not clear. Skinner et al. (2009) attempted to address this statistically, analysing chicken chromosomes that were outliers to the ‘small=more gene dense rule’. An alternative approach, however, is to analyse ‘former’ microchromosomes (i.e. those that have since fused in evolution to become part of a larger chromosome) such as those seen in *Falconiformes* (Damas et al. 2017; O’Connor et al. 2018b), which largely retain their inherent microchromosomal properties such as gene density, GC content, and recombination rate (Hillier et al. 2004).

Microchromosomes are thought to have originated ~ 400 mya (million year ago) in the ancestral vertebrate karyotype (Burt 2002). Bioinformatic reconstructions demonstrated that avian microchromosomes corresponded directly with gnathostome ancestor protochromosomes (Nakatani et al. 2007), suggesting that they have remained remarkably unchanged throughout evolution. Indeed, in a recent study, we suggested that the typical avian-like pattern became mostly established before birds and turtles diverged and was present in the theropod dinosaur lineage (O’Connor et al. 2018a). However, the lack of cytogenetic tools to characterize the microchromosomes described has impeded confirmation of this by direct evidence. The purpose of this study was therefore to test the hypothesis that inter-microchromosomal rearrangement is a rare phenomenon in avian evolution, i.e. that the genomic structure (karyotype) is widely stable (unchanged) across most species. Further, we hypothesized that fused ancestral former microchromosomes occupy a central nuclear position i.e. that their nuclear organization is a function of their genomic properties, not their physical size.

## Materials and methods

### Cell culture and chromosome preparation

Chromosome preparations were established from fibroblast cell lines generated from collagenase treatment of tracheal tissue or from skin biopsies. Cells were cultured at 40 °C and 5% CO_2_ in Alpha MEM (Fisher), supplemented with 10% foetal bovine serum (Gibco), 2% Pen-Strep (Sigma), and 1% l-glutamine (Sigma). Chromosome suspension preparation followed standard protocols: Briefly, mitostatic treatment with colcemid was performed at a final concentration of 5.0 μg/ml for 1 h at 37 °C, followed by 75 mM KCl hypotonic treatment for 20 min at 37 °C and fixation with 3:1 methanol/acetic acid. The species tested are listed in Table 1 and illustrated in Fig. 2.

**Table 1:** List of all avian species tested with the complete panel of microchromosome BACs

Order	Common name	Species name	2n
*Anseriformes*	Mallard	*Anas platyrhynchos*	80
*Charadriiformes*	Eurasian woodcock	*Scolopax rusticola*	96
*Columbiformes*	Rock dove	*Columba livia*	80
*Columbiformes*	Eurasian collared dove	*Streptopelia decaocto*	76
*Falconiformes*	Peregrine falcon	*Falco peregrinus*	50
*Falconiformes*	Saker falcon	*Falco cherrug*	52
*Falconiformes*	Gyrfalcon	*Falco rusticolus*	52
*Galliformes*	Turkey	*Meleagris gallopavo*	80
*Galliformes*	Chinese quail	*Coturnix chinensis*	78
*Galliformes*	Japanese quail	*Coturnix japonica*	78
*Galliformes*	Guinea fowl	*Numida meleagris*	78
*Galliformes*	Indian peafowl	*Pavo cristatus*	78
*Galliformes*	Sand partridge	*Ammoperdix heyi*	78
*Otidiformes*	Houbara bustard	*Chlamydotis undulata*	76
*Passeriformes*	Common blackbird	*Turdus merula*	80
*Passeriformes*	Atlantic canary	*Serinus canaria*	80
*Passeriformes*	Zebra finch	*Taeniopygia guttata*	80
*Psittaciformes*	Budgerigar	*Melopsittacus undulatus*	62
*Psittaciformes*	Cockatiel	*Nymphicus hollandicus*	72
*Psittaciformes*	Red-crowned parakeet	*Cyanoramphus novaezelandiae*	70
*Strigiformes*	Pharaoh eagle-owl	*Bubo ascalaphus*	72
*Struthioniformes*	Common ostrich	*Struthio camelus*	80

### Selection and preparation of BAC clones for FISH

Two BACs, selected from chicken galgal4 assembly (Hillier et al. 2004) and positioned as close as possible to each end of the chromosome, were chosen for each available reference microchromosome (GGA10-28 with the exception of GGA16) from the universal avian zooFISH probe set developed in our previous study by Damas et al. (2017) (Table S1). BAC clone DNA was isolated using the Qiagen miniprep kit prior to amplification and direct labelling by nick translation. Probes were labeled with Texas red-12-dUTP (Invitrogen) and FITC-fluorescein-12-UTP (Roche) prior to purification with the Qiagen nucleotide removal kit.

### FISH—metaphase analysis

Metaphase preparations were fixed to slides and dehydrated through an ethanol series (2 min each in 2× SSC, 70%, 85%, and 100% ethanol at room temperature). Probes were mixed with COT-1 DNA (Insight Biotech) and air-dried on to Cytocell octochrome (eight-chamber) and multiprobe (24-chamber) devices. Probes were subsequently rehydrated in formamide-based hybridization buffer before being aligned to the corresponding glass slide with fixed chromosome suspension on a 37 °C hotplate. Probe and target DNA were simultaneously denatured for 2 min on a 75 °C hotplate prior to hybridization in a humidified chamber for 72 h at 37 °C. Slides were washed post-hybridization for 30 s in 2× SSC/0.05% Tween 20 at room temperature and counterstained using VECTASHIELD anti-fade medium with DAPI (Vector Labs). Images were captured using an Olympus BX61 epifluorescence microscope with cooled CCD camera and SmartCapture (Digital Scientific UK) system. In order to exclude the possibility that there were any fusions or fissions between microchromosomes, a BAC from each microchromosome was tested with a BAC from all other remaining microchromosomes i.e. a BAC for GGA10 was tested individually with the entire set from GGA11 to GGA28.

### FISH—nuclear organization analysis

Selected BAC clones were pooled to maximize signal intensity in interphase nuclei (listed in Table 2). FISH and microscopy were performed as described for metaphase analysis but with single glass slides rather than multiple hybridization tools. Chromosome positioning was analysed using the ImageJ plugin Nuclear Morphology Analysis version 1.13.5 (https://bitbucket.org/bmskinner/nuclear_morphology/wiki/Home). At least 75 different nuclei were analysed for each chromosome. The positions of chromosome territories within the nucleus were assessed by measuring the proportions of FISH signal within concentric shells as previously described (Skinner et al. 2009). Signals were tested for significantly different distribution to a random pattern using a chi-square test. In addition, for each chromosome, images from the ‘unfused’ species were pooled for shell analysis. Chi-square tests were performed between the ‘fused’ and unfused groups, with Bonferroni correction for multiple testing.

**Table 2:** BAC combinations used to create probes for nuclear organization analysis, where set one corresponds to a fused microchromosome in falcons and set two corresponds to a fused microchromosome in budgerigar (position of the clones is listed in Table S1)

Probe set	BAC clone name	GGA Chr
Set one (fused micro in falcons)	CH261-10F1	19
	TGMCBA-356O18	
	CH261-50H12	
Set two (fused micro in budgerigar)	CH261-42P16	17
	TGMCBA-197G19	
	TGMCBA-375I5	

## Results

### Interchromosomal microchromosome organization

**Fig. 1:**
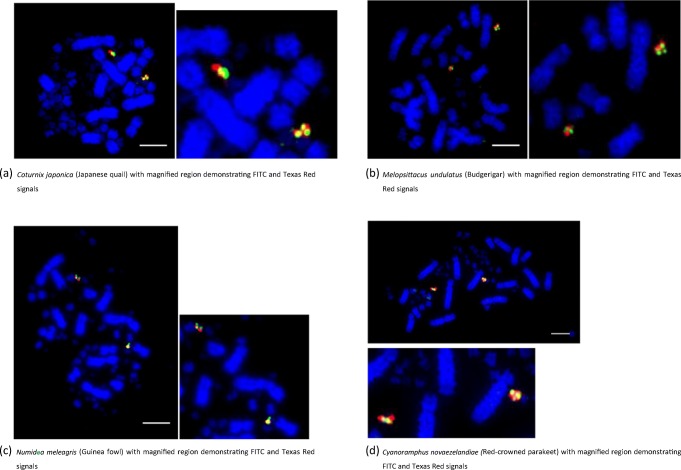
Probes for chicken chromosome 24 (CH261-103F4 FITC and CH261-65O4 Texas Red) tested on multiple avian species revealing no evidence of change from the pattern evident in chicken. Scale bar 10 μm. **(a)** Coturnix japonica (Japanese quail) with magnified region demonstrating FITC and Texas Red signals. **(b)** Melopsittacus undulatus (Budgerigar) with magnified region demonstrating FITC and Texas Red signals. **(c)** Numida meleagris (Guinea fowl) with magnified region demonstrating FITC and Texas Red signals. **(d)** Cyanoramphus novaezelandiae (Red-crowned parakeet) with magnified region demonstrating FITC and Texas Red signals

Results showed no evidence of inter-microchromosomal rearrangement in any of the orders analysed except *Falconiformes* and *Psittaciformes.* That is, clear punctate signals were achieved for all species with the exception of BACs for chicken chromosome 25 which did not hybridize on the zebra finch, blackbird and the canary. In addition, even in *Falconiformes* and *Psittaciformes*, chromosomes 22, 24, 25, 26 and 27 (numbers of chicken orthologues used for reference) appeared as similarly sized, entire microchromosomes. Examples are demonstrated in Fig. 1 for chromosome 24 in four species. In other words, the *Galliformes*, *Anseriformes*, *Charadriiformes*, *Columbiformes*, *Otidiformes*, *Passeriformes*, *Strigiformes* and the *Struthioniformes* all display the ancestral microchromosomal pattern. In fact, even in *Scolopax rusticola*, which exhibits an unusually high diploid number of 96, we do not find any evidence of microchromosomal fission, suggesting that in this case, the high diploid number is a result of macrochromosomal fission.

**Fig. 2:**
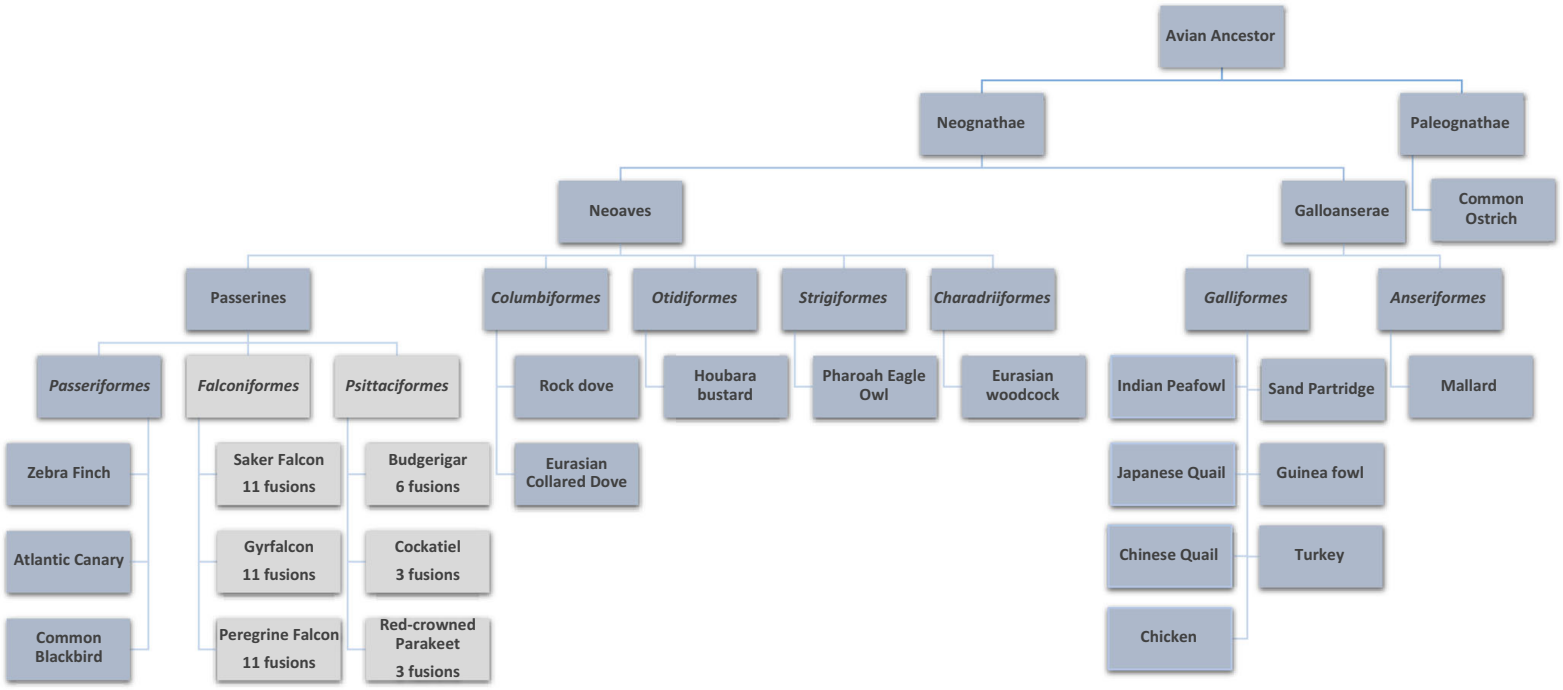
Tree (based on Jarvis et al. (2014) illustrating the lack of interchromosomal rearrangement of the microchromosomes. No interchromosomal microchromosome fusions from the avian ancestor unless otherwise stated (macrochromosomal fusions not listed). The overall pattern of microchromosome stability and rearrangement across the species is illustrated

**Fig. 3:**
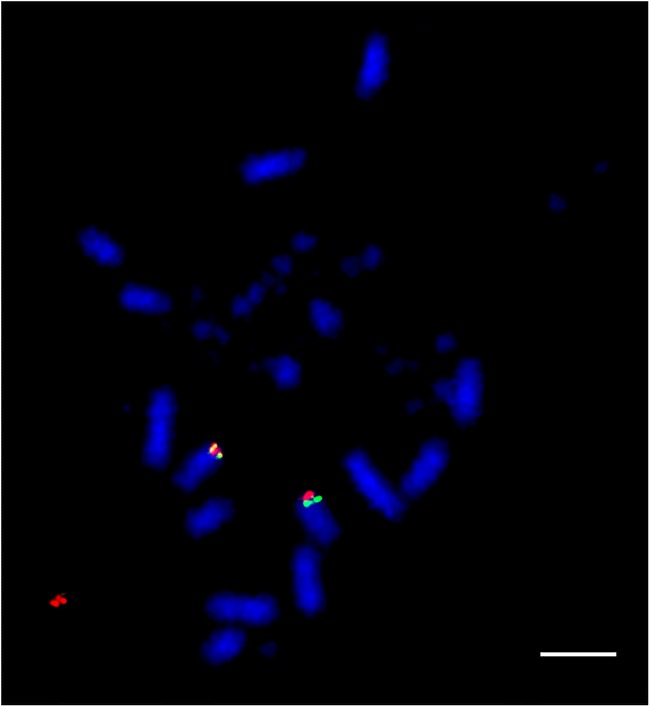
Hybridization of GGA11 BACs (CH261-121N21-FITC and CH261-154H1-Texas red) to cockatiel (*Nymphicus hollandicus*) metaphases illustrating fusion of ancestral microchromosome to a macrochromosome, subsequently revealed to be the homolog of GGA1. Scale bar 10 μm

Among the *Psittaciformes*, interchromosomal rearrangements were detected for the homologs for GGA10, 11, and 14 in all three species tested (Fig. 2). The red-crowned parakeet (*Cyanoramphus novaezelandiae*), the cockatiel (*Nymphicus hollandicus*), and the budgerigar (*Melopsittacus undulatus*) exhibit a fusion of each of these homologs to macrochromosomes, as illustrated in Fig. 3 where the homolog of GGA11 is shown fused to a macrochromosome in the cockatiel. The budgerigar also demonstrated fusions of GGA12, 13, and 17.

**Fig. 4:**
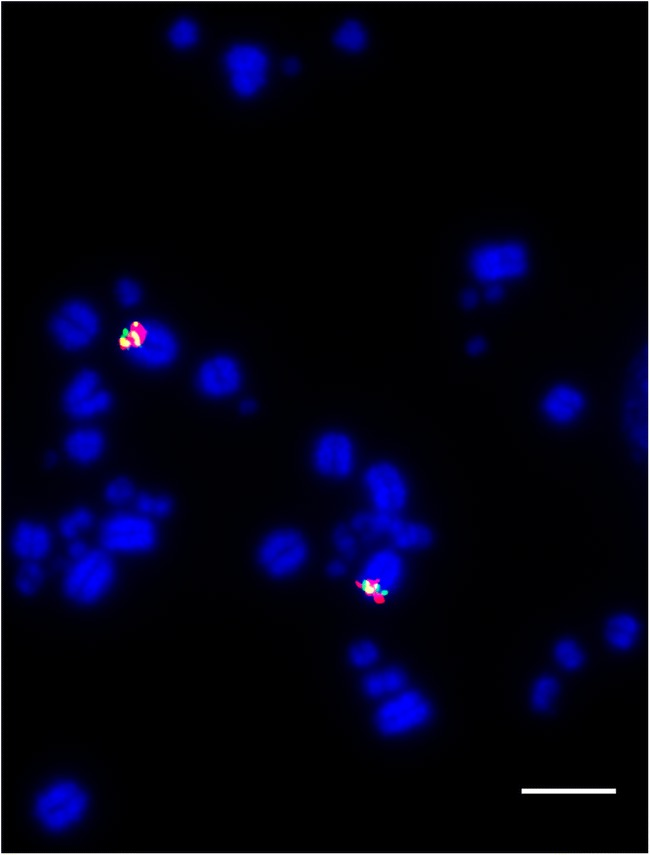
Hybridization of GGA18 BACs (CH261-60N6-FITC and CH261-72B18-Texas red) to saker falcon (*Falco cherrug*) metaphases illustrating fusion of ancestral microchromosome to a macrochromosome. Scale bar 10 μm

Among the *Falconiformes*, extensive rearrangement appears to have taken place with regions homologous to GGA microchromosomes 10, 12, 13, 14, 15, 17, 18, 19, 21, 23 and 28 fused to larger chromosomes. An example of which is illustrated in Fig. 4 where GGA18 homologs are fused to a macrochromosome in the saker falcon. Lineage-specific rearrangements were apparent with no evidence of chicken chromosome homologs 15, 18, 19, 23 and 28 being rearranged in any of the other (non-falcon related) species tested. Interestingly, 15, 18 and 19 appear to have fused together as one chromosome (to chicken homolog 4) in all three falcon species tested, while 23 and 28 have both fused to the homolog of chicken chromosome 2. All of the falcon species tested (peregrine, gyr and saker) appear to exhibit the same pattern of rearrangement (with the exception of peregrine chromosome 1) suggesting that the pattern was present in the ancestral falcon lineage. In addition, there appears to be no interchromosomal rearrangement between each pair of BACs tested, suggesting that, despite a fusion event occurring, these regions of DNA are highly conserved and not prone to breakage.

### Nuclear organization

**Fig. 5:**
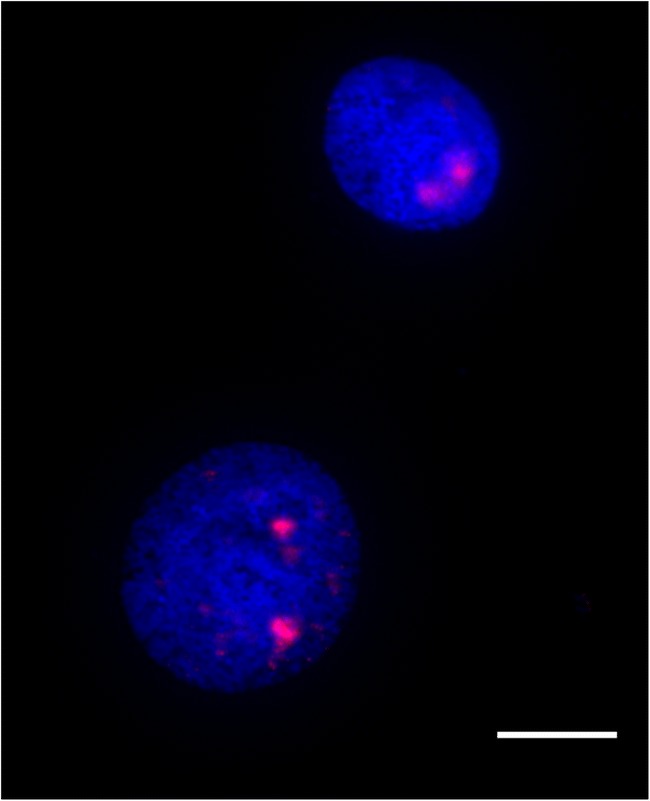
Example image of pooled BACs for GGA19 hybridized to interphase nuclei of the peregrine falcon (*Falco peregrinus*). Scale bar 10 μm

**Fig. 6:**
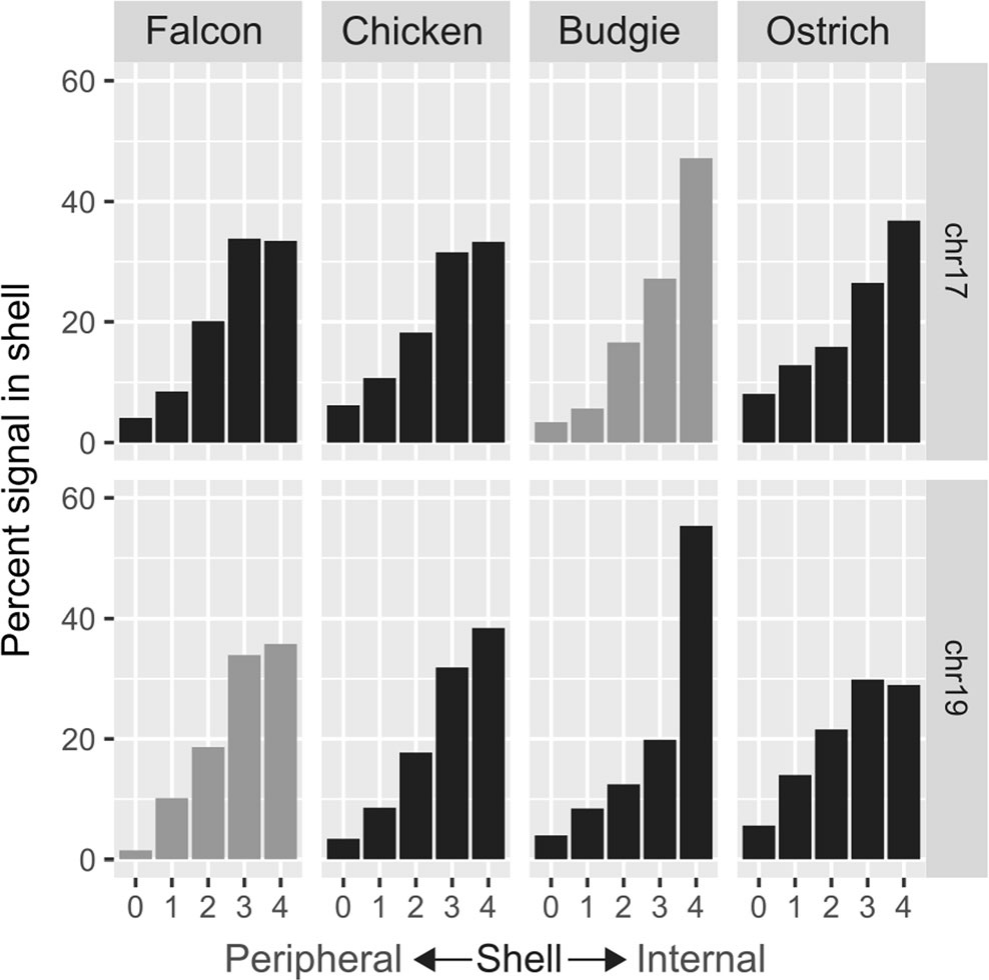
Localization of chicken microchromosome 17 and 19 probes in the nucleus of peregrine falcon, chicken, budgerigar, and ostrich where 0 represents the most peripheral region and 4 the most central nuclear region. Bars shaded in gray represent the species that demonstrates a fused microchromosome to a macrochromosome

We tested the hypothesis that the fusion of a microchromosome to a macrochomosome would cause the microchromosome territory to become more peripheral (i.e. that the position is driven by chromosome size, rather than sequence composition). Analysis suggests that genomic regions that were, ancestrally, microchromosomes occupy a central nuclear position. That is, when pooling BAC clones for macro and microchromosomes as detailed in Table 2, and hybridizing them to chicken (representing the unfused ancestral state), budgie (representing fusion of GGA3 and GGA17 and peregrine falcon (representing fusion of GGA4q and GGA19), and ostrich (representing a distant relative with the unfused ancestral state) patterns rarely differed regardless of whether the microchromosome was attached to a larger chromosome or not. We grouped the images for each chromosome (an example of which is demonstrated in Fig. 5) into fused and unfused pools, and tested for differences in distribution using a chi-square test. For GGA19, we find no significant difference (*p* = 0.82) between groups. For GGA17, there is a significant difference (*p* = 0.0077); the fused signal is more internal than the unfused signal, allowing us to reject the hypothesis that fusion of microchromosomes to macrochromosomes causes the microchromosome territory to migrate to a more peripheral location as demonstrated in Fig. 6.

The total nuclei with signals in the combined dataset are:

Peregrine Falcon chr17 set 178.

Peregrine Falcon chr19 set 189.

Chicken chr17 set 162.

Chicken chr19 set 183.

Budgerigar chr17 set 178.

Budgerigar chr19 set 110.

Ostrich chr17 set 178.

Ostrich chr19 set 159.

## Discussion

Results presented here demonstrate that the extraordinary degree of genome stability evident in the macrochromsomes of birds also extends to the (previously intractable) microchromosomes, illustrating a hitherto undiscovered overall genome stability (i.e. consistency of karyotype) that is rarely seen in other classes. As demonstrated in Fig. 2, our analysis of over 22 species across 10 avian orders reveals that 8 of the 10 orders demonstrate no change from the microchromosomal pattern seen in the chicken. Of the two orders where interchromosomal rearrangements had previously been identified using macrochromosome paints (*Falconiformes* and *Psittaciformes* (Nishida-Umehara et al. 2007; Nanda et al. 2007)), we have now been able to identify the ancestral microchromosomes involved in those rearrangements.

Of the avian species that exhibit microchromosomal rearrangements, the three representatives of the *Falconiformes* tested here (the saker, gyr and peregrine falcons) share the same pattern of fusion. This would suggest that the common ancestor of the falcons had the same karyotypic structure and that there has been little interchromosomal change since. Of the other highly rearranged order, the *Psittaciformes*, the microchromosomal fusions exhibited in each of the species tested differ from one another, suggesting that karyotypic evolution has continued from their common ancestor and that there are species-specific rearrangements. However, in all of these cases, it appears that there is a pattern of microchromosomes remaining as discrete units even when fused into highly complex karyotypes. Interestingly, this same pattern is also evident in the chicken, where the p-arm of chromosome four is a microchromosome in most other species, whilst retaining its uniquely microchromosomal characteristics such as high GC and gene content (Hillier et al. 2004).

Even taking into account the lineage-specific rearrangements, there appear to be five microchromosomes that across all birds tested, remain as microchromosomes with no signs of apparent fusion. In the chicken, these are five of the smallest sequenced chromosomes (GGA22, 24, 25, 26, 27) with sizes ranging from 4 to 6.5 Mb. Further sequence analysis may reveal signature features of these chromosomes that indicate a biological reason as to why these chromosomes are left intact. If there is any correlation with the size of the chromosomes and their lack of interchromosomal rearrangement, then this would suggest that the very smallest D-group chicken microchromosomes (Masabanda et al. 2004) which do not to date have sequences associated with them are also less prone to chromosomal fusion.

### Conservation of microchromosome synteny

The lack of interchromosomal rearrangement observed in this study either suggests an evolutionary advantage to retaining this signature avian configuration or else little opportunity for change. Evidence of a disproportionate amount of intrachromosomal change in pigeons (Damas et al. 2017) and *Passerines* (Skinner and Griffin 2011; Romanov et al. 2014; Zhang et al. 2014; Farré et al. 2016) suggests, however, that intrachromosomal change proceeds largely un-hindered and can accelerate in line with rapid speciation events. Indeed, the near absence of interchromosomal rearrangement is no barrier to diversity, and a direct correlation has been reported between the rates of speciation and intrachromosomal rearrangement (King 1995). There may even be an evolutionary advantage to maintaining a karyotypic structure formed of many compact, gene-rich microchromosomes (Romanov et al. 2014; O’Connor et al. 2018a). Burt (2002) suggested that a higher recombination rate contributed to the unique genomic features seen in microchromosomes such as high GC content, low repeats and high gene density which subsequently led to the maintenance of the typical avian karyotype. We recently reported the first emergence of this karyotypic structure for > 250 million years, before the divergence of birds and turtles and probably present in many dinosaur groups (O’Connor et al. 2018a). Reasons for its long-lived success are in the realms of speculation but might be due its ability, facilitated by many chromosomes, including microchromosomes with high recombination rates, to generate variation, which is thought to be the driver of natural selection. That is, a larger number of small chromosomes inherently generate variation through increased genetic recombination and increased random chromosome segregation. Variation, in turn, facilitates adaptation and may therefore have contributed to the wide phenotypic variation seen in birds and other dinosaurs.

### Conservation of nuclear organization

Our results also demonstrate that the highly stable genome organization of microchromosomes and macrochromosomes seen at metaphase is perhaps even more conserved at interphase, with each ancestral microchromosome preferentially locating in the centre. Remarkably, these microchromosomes still maintain their central position in the nucleus even when recently fused to a larger chromosome (as in the falcons and parrots). Particular loci are known to remain in ‘spatial synteny’; Véron et al. (2011) demonstrated that orthologous loci remained in three-dimensional proximity between human and mouse, despite the large karyotypic differences. Our results suggest the same to be true in birds.

This then raises the question of why does such nuclear organization persist, despite the karyotype being rearranged (and the microchromosomes now attached to larger ones)? The attachment of chromatin to the nuclear lamina is mediated, in mammals, by gene-poor, AT-rich elements known as Lamin Associated Domains (LADs) (Meuleman et al. 2013). Since microchromosomes tend to be both gene-rich and AT-poor, they may lack the necessary motifs to bind lamin proteins no matter what the karyotypic configuration. It is possible therefore that these motifs subsequently accumulate on fused microchromosomes; however, we would expect pressures against this: The internal gene-dense microchromosomes could provide access to transcription factories (Sexton et al. 2007) and keep genes safely away from the silencing environment of peripheral heterochromatin (e.g. Finlan et al. 2008). It is also possible that the macrochromosomes lose their lamin attachments; however, this also seems unlikely; modeling of chromatin dynamics suggests that the entire nuclear organization can invert when this tethering is interrupted (Falk et al. 2018) and that heterochromatin toward the centre is a default state which must be interrupted to achieve the mammalian (and avian) organization. Some chromatin must therefore remain tethered to the nuclear periphery, implying that macrochromosomal sequence will also be conserved. We propose that this model of nuclear organization represents a genomic configuration that has existed since at least early vertebrate evolution, and perhaps before.

Particular sequences have been found that mediate the interaction of chromatin with the lamina in mouse embryonic fibroblasts, correlating with CTCF binding site enrichment and local H3K27me3 and H3K9me2/3 methylation state (Harr et al. 2015). We expect that these features will show a conserved pattern in avian species also; CTCF enrichment has already been shown to correlate with the spatial organization of CpG islands in chicken lymphoid cells (Gushchanskaya et al. 2014). Furthermore, it should be noted that the probes used here were pooled BACs, not complete chromosome paints. If, as it seems, GC content and gene density is the driving factor for nuclear organization, we would expect a similar pattern at the sub-chromosomal level. That is, gene-dense regions of the chromosome should be more internal than gene-sparse regions of the chromosome. With the improvements to avian genome assemblies, we will soon be able to address these questions. As chromosome conformation data on birds accumulates, we predict that even in the most highly rearranged karyotypes, there will be a conserved proportion of sequence on the gene-poor chromosomes directing the overall architecture of the nucleus.

## Conclusions

The remarkable conservation of genome organization at a karyotypic level, and even more so at a nuclear level in birds (and possibly other dinosaurs (O’Connor et al. 2018a)) is a situation rarely reported in nature. It is certainly not the case in mammals, the most studied of clades, which display great variation in karyotypic and nuclear organization. Conservation for such a long time period implies evolutionary success, and we are aware of no other animal group for which this applies to such a degree.

## Electronic supplementary material

Table S1 (DOCX 21 kb)
