# Supplementary material for: Patterns of microchromosome organization remain highly conserved throughout avian evolution
Source: Chromosoma. 2018 Nov 17;128(1):21–9. doi: 10.1007/s00412-018-0685-6 (PMC6394684; doi:10.1007/s00412-018-0685-6)
Supplement: Supplementary file 1 — (DOCX 21 kb) [file 412_2018_685_MOESM1_ESM.docx]

**Supplementary Materials**

| **Clone Name** | **GGA Chrom** | **GGA Start** | **GGA End** | **GGA Span** | **GGA Chr Size** |
| --- | --- | --- | --- | --- | --- |
| CH261-115G24 | 10 | 11,912,518 | 12,133,656 | 221,139 | 20,435,342 |
| CH261-71G18 | 10 | 16,654,355 | 16,872,798 | 218,444 | 20,435,342 |
| CH261-121N21 | 11 | 8,706,813 | 8,955,579 | 248,767 | 20,218,793 |
| CH261-154H1 | 11 | 16,866,222 | 17,080,951 | 214,730 | 20,218,793 |
| CH261-60P3 | 12 | 12,115,391 | 12,258,173 | 142,783 | 19,948,154 |
| CH261-4M5 | 12 | 18,384,357 | 18,576,706 | 192,350 | 19,948,154 |
| CH261-115I12 | 13 | 14,037,105 | 14,230,515 | 193,411 | 18,407,460 |
| TGMCBA-321B13 | 13 | 14,903,339 | 15,053,072 | 149,733 | 18,407,460 |
| CH261-122H14 | 14 | 3,700,433 | 3,898,549 | 198,117 | 15,595,052 |
| CH261-69D20 | 14 | 12,492,480 | 12,679,652 | 187,173 | 15,595,052 |
| CH261-90P23 | 15 | 1,665,365 | 1,850,750 | 185,386 | 12,762,846 |
| TGMCBA-266G23 | 15 | 11,679,111 | 11,833,972 | 154,861 | 12,762,846 |
| TGMCBA-375I5 | 17 | 3,741,293 | 3,870,581 | 129,288 | 10,956,400 |
| CH261-42P16 | 17 | 9,697,682 | 9,869,418 | 171,737 | 10,956,400 |
| CH261-60N6 | 18 | 4,427,448 | 4,660,385 | 232,938 | 11,053,727 |
| CH261-72B18 | 18 | 10,844,632 | 11,016,876 | 172,245 | 11,053,727 |
| CH261-10F1 | 19 | 1,396,201 | 1,536,729 | 140,529 | 9,979,828 |
| CH261-50H12 | 19 | 7,456,038 | 7,607,889 | 151,852 | 9,979,828 |
| TGMCBA-250E3 | 20 | 421,458 | 570,315 | 148,858 | 14,109,371 |
| TGMCBA-341F20 | 20 | 10,890,041 | 11,017,721 | 148,858 | 14,109,371 |
| CH261-83I20 | 21 | 2,221,879 | 2,415,984 | 194,106 | 6,862,722 |
| CH261-122K8 | 21 | 3,750,054 | 3,931,234 | 181,181 | 6,862,722 |
| CH261-40J9 | 22 | 2,040,842 | 2,218,076 | 177,235 | 4,729,743 |
| CH261-18G17 | 22 | 2,462,685 | 2,677,143 | 214,459 | 4,729,743 |
| CH261-191G17 | 23 | 8,882 | 226,490 | 217,609 | 5,786,528 |
| CH261-90K11 | 23 | 5,462,306 | 5,624,399 | 162,094 | 5,786,528 |
| CH261-103F4 | 24 | 1,498,400 | 1,650,351 | 151,952 | 6,280,547 |
| CH261-65O4 | 24 | 3,204,248 | 3,355,883 | 151,636 | 6,280,547 |
| CH261-59C21 | 25 | 1,055,115 | 1,210,165 | 155,051 | 2,906,300 |
| CH261-127K7 | 25 | 1,097,735 | 1,222,152 | 124,418 | 2,906,300 |
| CH261-186M13 | 26 | 1,602,123 | 1,778,765 | 176,643 | 5,313,770 |
| CH261-170L23 | 26 | 3,746,540 | 3,941,395 | 194,856 | 5,313,770 |
| CH261-66M16 | 27 | 4,854,099 | 5,031,264 | 177,166 | 5,655,794 |
| CH261-28L10 | 27 | 5,232,465 | 5,448,863 | 216,399 | 5,655,794 |
| CH261-64A15 | 28 | 3,706,734 | 3,876,730 | 169,997 | 4,974,273 |
| CH261-72A10 | 28 | 4,386,181 | 4,596,229 | 210,049 | 4,974,273 |

Table S1: BACs chosen for multiple cross species analysis with corresponding positions in the chicken genome (galgal4). Two BACs were chosen from either the chicken CHORI-261 library or the zebra finch TGMCBA library for each chromosome using the chicken genome as the reference. Size of each chicken microchromosome and span of each BAC is also listed to indicate position of the BACs relative to each chromosome.
